# An electronic product carbon footprint dataset for question answering

**DOI:** 10.1038/s41597-026-06544-5

**Published:** 2026-01-14

**Authors:** Kaiwen Zhao, Ajesh Koyatan Chathoth, Bharathan Balaji, Stephen Lee

**Affiliations:** 1 University of Pittsburgh, Pittsburgh, PA USA; 2 Amazon, Seattle, WA USA

**Keywords:** Environmental impact, Environmental sciences

## Abstract

The embodied carbon of computing systems constitutes a significant portion of their greenhouse gas (GHG) emissions. To support environmental initiatives and meet evolving standards, many companies now disclose product carbon footprints in sustainability reports, often with detailed breakdowns. Yet these reports appear in diverse and unstructured formats—text, tables, and graphs embedded in PDFs—creating major challenges for extracting and analyzing component-specific emissions data. This lack of standardization limits comparative assessments and opportunities for targeted reductions. To address this, we introduce a carbon question-answering (QA) dataset designed to enable the extraction and analysis of data from carbon reports of computing products. The dataset features annotated metadata, numerical reasoning tasks, and structured derivations to ensure accurate processing of fragmented information. Because approximately 75% of products in the dataset follow the PAIA (MIT) model for carbon footprinting, the dataset primarily reflects PAIA-style reporting practices, offering insight into how industry methods influence reported values. This work establishes a foundation for training advanced language models to automate aggregation and standardization of emissions data for ICT systems.

## Background & Summary

With the growing demand for computing, the environmental sustainability of information and communication technology (ICT) systems has become a critical concern^1^. Emissions from computing infrastructure currently comprise a significant portion of their carbon footprints^2^. Recent studies estimate that ICT systems account for around 3% of global operational emissions^3^. Although research has primarily focused on operational emissions, much less attention is paid to the embodied emissions of ICT technologies, generated during the manufacturing of these systems^3^. Thus, there is an urgent need to account for and address the carbon footprint of ICT manufacturing^4^.

To address this issue, recent efforts have focused on developing tools that provide detailed insights into the carbon footprints of ICT products^5–8^. For example, the ACT tool offers a comprehensive breakdown of ICT carbon footprints, enabling carbon-aware exploration and optimization of various systems. These tools enable users to analyze various strategies for reducing emissions and improving sustainability^5^. However, their accuracy depends on the quality of the underlying carbon footprint data. This data is usually sourced from sustainability reports, which often lack standardization and are frequently presented in unstructured data formats, such as text or graphical data embedded in Portable Document Format (PDF) documents. These inconsistencies make it difficult to extract and analyze component-level emissions, requiring significant manual effort to reconcile and interpret the information. There are also tools independent of sustainability reports, such as the imec.netzero webapp^9,10^ (https://netzero.imec-int.com/), which quantifies integrated circuit (IC) manufacturing footprints using bottom-up process modeling. However, a bottom-up approach requires process-level data from manufacturers, which is often not publicly available.

The above challenges highlight the need for standardized and comprehensive quantitative datasets on embodied greenhouse gas (GHG) emissions across ICT components. Recent advancements in large language models such as GPT-4, Llama 3, and Gemini^11–13^ create new opportunities to address this gap. These models are capable of performing question-answering (QA) tasks that involve numerical reasoning and extracting relevant data from unstructured documents, which could enable the creation of standardized emission datasets^14–18^. For example, studies have shown that LLMs can automate the processing of fragmented and inconsistent information, reducing the manual effort required to reconcile diverse reporting formats. However, the effectiveness of such models requires well-annotated training datasets, which serve as the foundation for their ability to reason effectively and generate reliable outputs^19^.

**Fig. 1:**
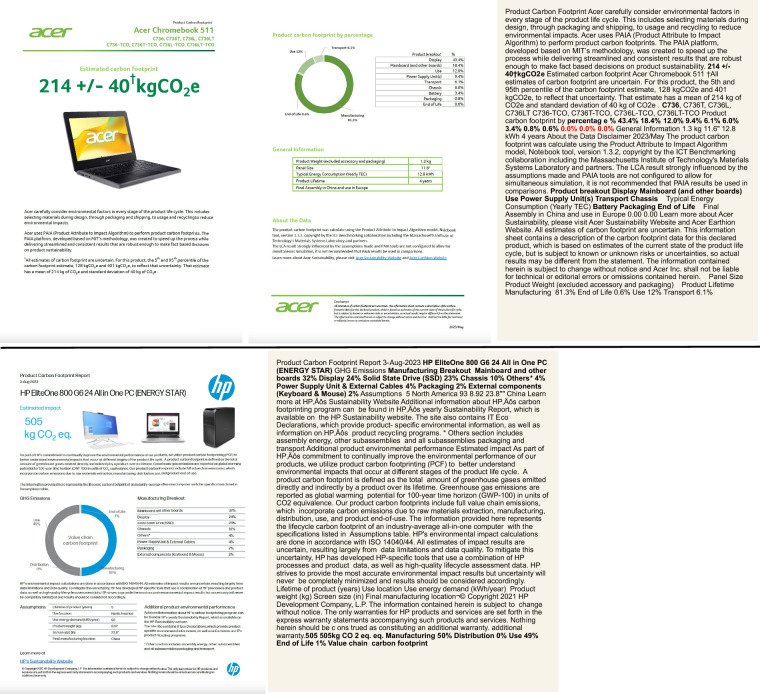
Acer and HP PCF report and extracted text examples.

We present the first carbon QA dataset designed to extract data from real-world carbon reports of products from various companies. This dataset enables the development of models that can facilitate the extraction and standardization of carbon data from various unstructured reports. In Table 1, we compare our dataset with existing QA benchmarks as well as the non-QA PCF dataset Boavizta (https://github.com/Boavizta/environmental-footprint-data). Unlike most datasets, which focus exclusively on text or well-structured tables, our dataset also incorporates text extracted from charts. A key distinction of our dataset, unlike prior benchmarks that are typically clean and well-formatted, is that our tables are extracted directly from PDFs and often contain inconsistencies. As shown in Fig. 1 of Acer (https://www.acer.com/us-en/sustainability/product-carbon-footprint) and HP (https://h20195.www2.hp.com/v2/) example reports, rows from the same table may appear fragmented or overlap across paragraphs, and related values are frequently split across sections. For example, the total footprint may appear on one page and the component breakdown in a table elsewhere. Although tabular data looks structured visually, PDFs do not encode it as true tables, and hidden or misaligned text further complicates extraction. PCF-QA dataset, which includes ground-truth annotations, provides a foundation for developing QA techniques that can robustly handle noisy, fragmented, and heterogeneous numerical text. The Boavizta dataset also collected environmental data from Product Carbon Footprint (PCF) reports, covering a total of 11 companies, including Apple and Samsung. In our work, we aimed to explore carbon modeling with a stronger emphasis on arithmetic reasoning. Therefore, the manufacturing-stage carbon footprint breakdown provides an ideal foundation, which is collected for 1,735 products. In contrast, Boavizta includes only partial manufacturing breakdown data for approximately 10 products. Boavizta primarily extracted total product carbon emissions and value-chain breakdown percentages (manufacturing, transportation, use, and end-of-life), along with selected specifications such as reporting date, product lifetime, assembly and use locations, and screen size when available. However, we additionally collected information on the product carbon footprinting model or standard used—an element absent from Boavizta but crucial for interpreting product-level carbon results. Finally, our dataset uniquely provides a question-answer (QA) benchmark built on these reports, including detailed question types, evidence, executable reasoning programs, and verified answers. These components are key enablers for advancing future research on model training and evaluation in carbon reasoning.

**Table 1:** QA dataset comparison.

Dataset	# Doc.	Data Source	Content	Q. Type
SQuAD^24^	736	Text	Wikipedia	RC.
HybridQA^25^	13k	Text,Table	Wikipedia	RC.
TabFact^26^	16k	Text,Table	Wikipedia	FV.
TAT-QA^27^	182	Text,Table	Financial report	RC., AR.
Boavizta	1,226	Text,Table	Carbon report	N/A
**PCF-QA**	**1,735**	**Text,Table,Chart**	**Carbon report**	**RC., AR**.

Q. = Question, RC. = Reading Comprehension, FV. = Fact Verification, AR. = Arithmetic Reasoning. For HybridQA and TabFact, the document count refers to the number of tables.

Furthermore, the focus on product carbon reports opens new opportunities for advancing QA research in sustainability and environmental data processing. However, we found that many companies, including those in our dataset, often do not disclose the underlying carbon modeling methodologies used to calculate reported values. When available, we extract information about the methodology. For example, we capture the use of the Product Attributes to Impact Algorithm (PAIA)^20^ or other standards applied to compute the carbon footprint. Our dataset also captures scope-related features, such as assembly location, use location, and other scope-specific details, extracting them whenever available. Including this information increases transparency and supports more accurate environmental assessment.

To extract the above data, we develop custom Python program-based parsers that reliably extract values from carbon reports. Human annotators then validate this extracted data to ensure accuracy and reliability. Our dataset supports multiple use cases. It can be integrated with carbon sustainability tools, such as ACT^5^, which require component-level information to compute emission footprints. It also enables the development of models capable of predicting product-level carbon footprints and estimating contributions from individual components. The dataset includes annotated question-answer (QA) pairs that facilitate the design of robust QA systems capable of extracting and reasoning over emissions data. Such QA-based data extraction systems can further support the standardization and improved usability of sustainability-related information. It can provide alternatives in terms of reporting procedure formats that may lead to better data quality, transparency, and maintenance over time. In addition, program scripts are provided for each QA pair that can support research on program-based reasoning^21^, where models can perform multi-step calculations and logical operations directly on structured and semi-structured report data.

Note that our dataset represents a snapshot in time, and the accompanying scripts remain useful for parsing existing reports that follow the same reporting format. However, if the formatting of future product reports evolves, new scripts may be required to extract the data accurately. At present, the dataset focuses exclusively on carbon footprint data, as companies typically provide detailed product-level emissions. Other sustainability dimensions, such as water consumption or biodiversity loss, are generally not reported. Nevertheless, our data processing framework could be extended to incorporate these additional indicators if such data becomes available in the future. Despite this, the dataset provides a comprehensive and high-quality foundation for analyzing component-level emissions and building robust QA systems. Our key contributions are as follows:

- **CarbonPCF-QA Dataset:** The dataset contains 1,735 carbon reports of computing products from four companies. This dataset is valuable for training language models to perform numerical reasoning, providing carbon breakdown estimates for different components of a product, and enabling comparisons across products from different manufacturers.
- **Question-Answer Pairs:** The dataset includes various question-answer pairs, where the answers to these questions can serve as inputs to various sustainability tools. There are four types of numerical reasoning questions — namely, word match, max/min, top-k, and calculation – designed to evaluate the reasoning capabilities of models. For questions requiring calculations, we also provide annotated Python derivations that can generate the final answer. This is particularly useful for training models to handle complex numerical reasoning tasks to help the model learn these derivations.
- **Annotated Metadata:** The dataset also includes additional annotated metadata that specifies the source locations of the answers within the PDF documents, providing additional evidence to support the extracted answers. The dataset further includes metadata on product use location, assembly location, and the footprinting model/standard to track scope differences to improve clarity and comparability.
- **Analysis:** We also analyze and compare the carbon footprints of various products and their components. This analysis provides valuable insights that can guide policies for reducing embodied emissions in ICT systems.

## Methods

**Fig. 2:**
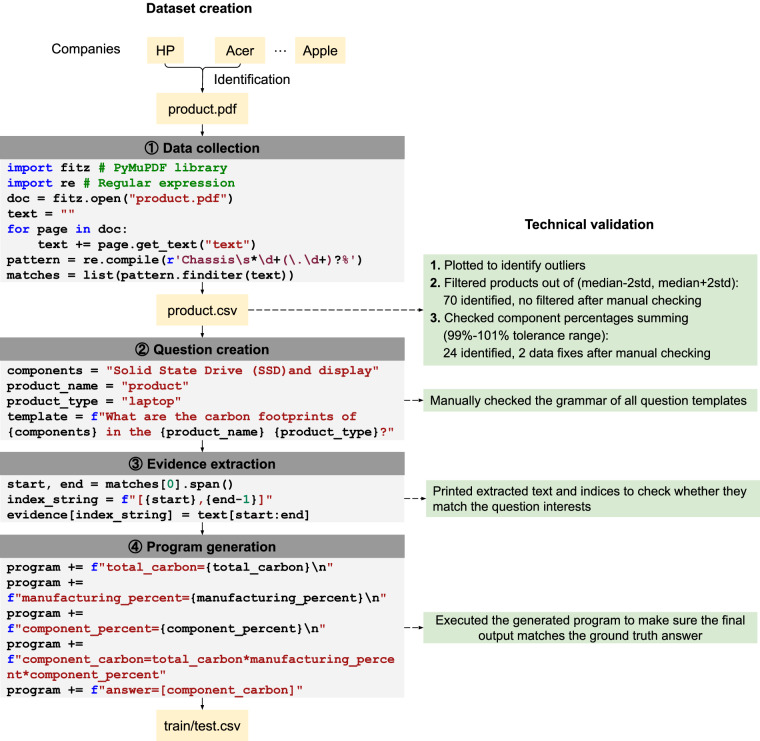
Our dataset creation and validation process. (**1**) **Data collection**: Values are extracted using regular expressions and then validated by human annotators. (**2**) **Question creation**: Standardized templates are used to generate question-answer pairs. (**3**) **Evidence extraction**: Scripts identify the extracted text and their indices and store them for reference. (**4**) **Program generation**: Python scripts are created using templates that execute the necessary calculations to produce the final answers.

Figure 2 illustrates the end-to-end workflow for constructing our dataset. We first identify suitable companies, such as HP and Acer, based on report availability and format consistency. Next, we collect Product Carbon Footprint (PCF) reports from company websites using automated scripts. We developed scripts to automatically download these reports and employed the PyMuPDF library (https://pymupdf.readthedocs.io/en/latest/) to convert the PDFs into editable raw text. Regular expression-based programs were then written to extract structured data entries from the text. During data cleaning, we performed technical validation by plotting the extracted values to identify outliers. Specifically, we removed products whose values fell beyond two standard deviations from the median, as well as cases where the sum of component percentages fell outside the tolerance range. This yielded our product-level dataset, *products.csv*. From this dataset, we generated question-answer (QA) pairs from the curated data using multiple templates tailored to different question types. Each generated question was manually reviewed for grammatical correctness and naturalness. In the following stage, we extracted supporting evidence directly from the PCF reports to construct accurate answers. To ensure correctness, we printed the extracted text along with character indices and verified that each piece of evidence was properly tagged. Finally, for each question, we created templates to generate executable programs that incorporated the extracted evidence and detailed derivation steps for computing the final answer. We executed these generated programs and confirmed that their outputs matched the ground-truth answers for validation. This procedure produced our QA datasets, *train.csv* and *test.csv*. Each step of this process is described in detail below.

### Data collection

We construct our dataset using product carbon footprint reports of computing products, summarized in Table 2. We collected 1,735 PDF reports from the websites of HP, Dell (https://www.dell.com/en-us/lp/dt/product-carbon-footprints), Acer, and Lenovo (https://www.lenovo.com/us/en/compliance/eco-declaration/). These four companies were chosen because they publish a large number of product carbon footprint (PCF) reports in a consistent format over multiple years, enabling automated parsing and structured data extraction. In contrast, reports from many other ICT companies, such as Apple (https://www.apple.com/environment/), vary in format across years. Other companies provide only a limited number of reports or present data mainly in visual forms like bar or pie charts, which makes the collection more difficult. Due to the manual effort required to extract and validate data from such reports, these companies were not included in our dataset. Each file averages approximately 4,000 characters and spans around two pages. Using the Python PyMuPDF library, we parsed and converted the PDF files into text for processing. To handle the diversity in formatting and content across reports, we developed custom parsers in Python tailored to extract relevant information accurately. These parsers are designed to identify and retrieve carbon-related data, including the total product carbon footprint (PCF) and its percentage breakdown across various manufacturing components. Given the variability in report formats between companies, we implemented multiple parsing strategies to account for these differences. Regular expressions were a core part of the parsing process, allowing us to match specific text patterns and locate key data within the converted text. This structured approach ensures reliable extraction of values and metadata, enabling us to create a consistent and comprehensive dataset from unstructured PDF reports.

**Table 2:** Summary statistics of PCF-QA dataset.

Statistics	Total
Number of Companies	4
Number of Files	1,735
Average number of characters per file	3,752
Average number of words per file	538
Average number of pages per file	1.76

The upper panel of Fig. 1 presents an Acer example, showing the original PDF document alongside the corresponding extracted raw text. These examples highlight the diversity of formats encountered in the reports and illustrate the extraction process. For instance, in one document format (e.g., Acer), the component names and their percentage values appear separately, as shown in Fig. 1. To systematically extract values, we implement a custom parser tailored to this specific PDF report template, leveraging regular expressions (regular expression). These regular expression patterns are designed to identify word patterns and their occurrences within the PDF and extract associated values. The regular expression patterns are crafted to uniquely detect components and their corresponding values, minimizing the risk of mismatches. A simplified example in Table 3 shows how the parser works. For instance, the parser identifies the occurrence of “Display” within the reference text and records its start and end indices, such as [0, 6]. This information allows the parser to locate and isolate component names accurately. Simultaneously, regular expression patterns tailored to detect numeric percentages extract all associated values. By combining the positional information of components with the extracted percentage values, the parser matches components to their corresponding values. For example, the parser pairs “Display” with “19.8%” by aligning their positions within their respective lists. This approach ensures accurate and reliable extraction while preserving the natural order of the data in the document. In cases where the structure deviates from expectations or new patterns emerge, a manual review is conducted to refine the regular expression patterns and improve parser robustness. This iterative refinement ensures the parser remains adaptable to variations in templates while maintaining high accuracy.

**Table 3:** An example showcasing the different attribute values, including question, PCF reference text, evidence extraction, program generation, and ground truth answers for calculation-based question types.

Attribute	Value
Question	What is the carbon footprint of the display in the laptop?
PCF text	... Display Batteries Chassis 19.8% 3.9% 2.2% ...
Evidence index and text	{... “[0, 6], [26, 30]”, “Display, 19.8%” ...}
Program	total_carbon=184 manufacturing_percent=0.79 display_percent=0.198 display_carbon=total_carbon *manufacturing_percent*display_percent answer=[display_carbon]
Ground truth answer	[28.78]

In another document format (e.g., HP, Dell), the breakdown is structured as a continuous sequence of {component name, numeric percentage, '%'} rather than being split across different sections. For instance, in the bottom panel of Fig. 1, the manufacturing breakdown follows the phrase “Manufacturing Breakout,” followed by each name-value pair separated by a ‘%’ symbol. Thus, we designed a custom regular expression that was specifically tailored to detect this format. The regular expression pattern is configured to identify component names based on their proximity to numeric percentages and the ‘%’ symbol, ensuring precise association between components and their respective values. For instance, in Fig. 2①, our script first uses the PyMuPDF library to open the product PDF file as a raw text string. We then apply a regular expression to detect strings such as “Chassis” followed by a number (e.g., 8) and a percentage symbol (‘%’). Similarly, total product carbon footprint (PCF) values are identified using the suffix “kg CO2 eq.” Once detected, the parser extracts the data and stores it in a CSV file. As before, if the regular expression encounters a format deviation or ambiguity, manual reviews are conducted to validate the data and refine the extraction rules.

After the data collection, we validated the product carbon footprint (PCF) of each product for each company. We calculated the median and standard deviation of PCF for each company and filtered out products whose PCF values deviated by more than two standard deviations from the median. This filtering process identified 70 products, which we subsequently reviewed manually to ensure that the extreme values were not due to parsing errors. Upon manual verification, we confirmed that all values matched those reported in the original documents. Most of the large PCF values were associated with workstations, as they typically have more components with significantly higher carbon footprints due to energy consumption and cooling during the use phase, as well as a larger manufacturing carbon footprint. In contrast, the small PCF values were primarily attributed to tablets, which generally have fewer components and lower overall carbon footprints.

### Question creation

We design four distinct types of numerical reasoning questions for querying the carbon reports. This includes word matching, max/min identification, top-k ranking, and calculation-based derivations, as detailed in Table 4. Word matching questions involve extracting answers directly from the PDF without requiring calculations, such as the total carbon footprint or the percentage contributions of specific components. These questions primarily test the ability to locate and retrieve exact matches from the text. Max/min questions focus on identifying the components with the maximum or minimum carbon footprints, such as determining the highest or lowest contributor to overall emissions and evaluating the system’s capacity for numerical comparison. Top-k questions require identifying the top-ranked components based on their carbon footprints, such as the top three contributors. Finally, calculation-based questions involve performing arithmetic operations to derive answers, such as calculating the combined carbon footprint of multiple components or determining the percentage contribution of a subset to the total footprint. Example questions are shown in Table 5.

**Table 4:** Distribution of question types in PCF-QA dataset.

Question Type	Definition	Train	Test
Word Match	Direct extraction of values from text.	6,400	1,500
Max/Min	Identify the largest or smallest contributor.	1,920	450
Top 3/5	Rank and select the top components by footprint.	1,280	300
Calculation	Perform arithmetic to derive combined values.	5,192	1,238
Total	—	14,792	3,488

**Table 5:** Question and program examples of different question types in the dataset.

Question Type	Question	Program
Word Match	What are the carbon footprint percentages of the mainboard, chassis, and battery in the C736 laptop?	mainboard_percent=18.4 chassis_percent=6.0 battery_percent=3.4 answer=[mainboard_percent, chassis_percent, battery_percent]
Max/Min	What is the component with the highest carbon footprint percentage in the manufacturing breakdown of the C736 laptop?	breakdown_dict={“display”:43.4, “mainboard”:18.4, “chassis”:6.0, “battery”:3.4,“power”:9.4, “packaging”:0.8} max_pair= max(breakdown_dict.items(), key=lambda item: item[1]) answer=[max_pair[0]: max_pair[1]]
Top 3/5	What are the top 5 components with the highest carbon footprint percentages in the manufacturing breakdown of the C736 laptop?	breakdown_dict={“display”:43.4, “mainboard”:18.4,“chassis”:6.0, “battery”:3.4,“power”:9.4, “packaging”:0.8} answer = [k: v for k, v in sorted(breakdown_dict.items(), key=lambda item: item[1], reverse=True)[:5]]
Calculation	What is the carbon footprint of battery in the C736 laptop?	total_carbon=214 battery_percent=0.034 battery_carbon=total_carbon *battery_percent answer=[battery_carbon]

The questions are asked about Fig. 1 Acer report.

We employ various question templates to generate queries that iterate over the components extracted during the data collection process. A sample template for the calculation question (Fig. 2②) is: “What are the carbon footprints of {components} in the {product name}{product type}?” Here, placeholders such as {components} are dynamically replaced with specific component names (e.g., Solid State Drive (SSD), display), while {product name} and {product type} are substituted with details like product names and types (e.g., laptop, workstation) extracted from the PDFs. Similarly, we use other question templates for each question type to generate specific queries based on the content of the document. On average, at least 14 questions are generated per PDF, ensuring thorough coverage of the available data. Every question is designed to be answerable using information extracted directly from the corresponding PDF document. The ground truth answers are obtained from the structured data in the CSV file of collected data, resulting in a set of question-answer pairs.

### Evidence extraction

In the dataset, each question is linked to the specific location of the relevant text within the reference document necessary to answer it. This approach addresses the potential presence of spurious or duplicate text caused by errors in PDF extraction. For example, the parser might incorrectly extract text as “705 705kg CO2 eq. eq.” instead of “705kg CO2 eq.” due to inconsistencies in the PDF format. To address such issues, we treat the entire reference text as a character array and use the array indices to pinpoint the exact location of the supporting evidence. By leveraging these indexed positions, we can reliably extract only the relevant information, referred to as evidence, while discarding extraneous or duplicated text. The extracted evidence is then organized into a structured JSON format. Each question is associated with a JSON object, where the keys represent the start and end indices (Fig. 2③) of the evidence within the character array, and the values contain the corresponding extracted text. This evidence can be used as input to guide models in generating accurate answers to the questions or to validate their outputs against the extracted reference text.

### Program generation

The dataset includes a Python program specifically designed to generate outputs for each question. This program consists of a series of assignment steps that outline the logic for deriving the answer and may include simple arithmetic operations to compute the final results. This is similar to prior work that shows how program-aided derivations improve the numerical reasoning capabilities of models^21^. We generate the program by designing a specific template for each question type and filling it with values pulled from the CSV files. The templates (Fig. 2④) are intentionally kept simple, consisting of basic variable assignments, and do not rely on any external libraries. Moreover, variable names follow the convention {name}_{carbon/percent}, where {name} corresponds to the total PCF, manufacturing footprint, or component names, and the suffix indicates whether the variable represents a carbon footprint (‘carbon’) or a percentage (‘percent’). For questions that require multiple answers (e.g., carbon footprints for both an HDD and a chassis), the program structures the final answer as a list, with the components arranged in the order they appear in the question. Example programs are shown in Table 5.

For example, in the case of word match questions, such as those asking for the overall carbon footprint, we extract the relevant data from the evidence and assign it to a variable, which is then returned as the final answer. Similarly, for min-max questions, the template includes logic for identifying the minimum or maximum values from the list of component carbon footprints extracted from the evidence. The program then performs the appropriate arithmetic operations, such as taking the minimum or maximum, to compute the answer. However, for top-3 or top-5 questions, the program template sorts the list of component carbon footprints in descending order and extracts the top three or five values, depending on the question. It then returns these top values as a list as the final answer. For calculation questions, such as determining the carbon footprint of the chassis, we extract the total PCF along with the manufacturing and chassis percentages from the evidence. The carbon footprint of the chassis is then calculated by multiplying the total PCF by the respective percentages, providing the final answer. The programs for calculation questions vary depending on the reporting style. HP reports provide component percentages relative to the manufacturing carbon footprint, while Dell and Acer reports base them on the PCF. This leads to different methods of calculating component carbon footprints: for HP, the component footprint is calculated by applying the percentage to the manufacturing footprint, whereas for Dell and Acer, it is computed by multiplying the component percentage by the PCF. The program templates rely on simple operations: assignment for Word Match questions; max({*x*_*i*_}) or min({*x*_*i*_}) for Max/Min questions; sorting {*x*_*i*_} to return the top 3 or 5 components for Top-k questions; and assignment, addition, and multiplication

## Data Records

Our dataset is available on Figshare^22^. Tables 6 and 7 provide an overview of the dataset’s fields, sources, and types. The dataset consists of two types of CSV files: a Product CSV file in the product folder, which contains data extracted from PDF product carbon reports during the data collection step, and question-answer (QA) CSV files in QA folder, which include the generated question-answer records. We define the product ID, an incrementing value starting from 1, as the primary key for each product in the Product CSV file and use it as a foreign key in the QA CSV file. The product records contain fields such as product name and total product carbon footprint (PCF) that are directly extracted from the reports. We provide the file URL that directly links to the PDF report, along with an archive URL as a backup in case the file URL becomes inaccessible using WayBackMachine^23^ (https://github.com/agude/wayback-machine-archiver). Our dataset includes metadata fields for Use location, Assembly location, and the Product carbon footprinting model/standard. These additions capture scope-related differences and enhance transparency for end-users. Moreover, we collect manufacturing and component percentages, which vary by company. The dataset also contains a validation notes column, which provides information about the validation process for each data record, such as whether it was validated programmatically or manually. We divide the dataset into a training set and a test set, with an 80/20 split based on the documents. An example record from the content of the products.csv file is shown in Table 9.

**Table 6:** Data record glossary for PCF-QA dataset (products.csv).

Field	Source	Type
Product name	RD.	Text
File URL	RW.	Text
Archive URL	RW.	Text
Company name	RD.	Text
Product type	RD.	Text
Use location	RD.	Text
Assembly location	RD.	Text
Product carbon footprinting model/standard	RD.	Text
Product carbon footprint (PCF, kg CO2e)	RD.	Numeric
Manufacturing CO2e percentage	RD.	Numeric
Chassis & assembly CO2e percentage	RD.	Numeric
HDD CO2e percentage	RD.	Numeric
SSD CO2e percentage	RD.	Numeric
Power supply unit CO2e percentage	RD.	Numeric
Battery CO2e percentage	RD.	Numeric
Mainboard and other boards CO2e percentage	RD.	Numeric
Display CO2e percentage	RD.	Numeric
Packaging CO2e percentage	RD.	Numeric
ODD CO2e percentage	RD.	Numeric
External components CO2e percentage	RD.	Numeric
Others* CO2e percentage	RD.	Numeric
Manufacturing CO2e	CP.	Numeric
Chassis & assembly CO2e	CP.	Numeric
HDD CO2e	CP.	Numeric
SSD CO2e	CP.	Numeric
Power supply unit CO2e	CP.	Numeric
Battery CO2e	CP.	Numeric
Mainboard and other boards CO2e	CP.	Numeric
Display CO2e	CP.	Numeric
Packaging CO2e	CP.	Numeric
ODD CO2e	RD.	Numeric
External components CO2e	RD.	Numeric
Others* CO2e	RD.	Numeric
Validation notes	AD.	Text

PK. = Primary key. FK. = Foreign key. RD. = Raw data in the report. RW. = Raw data from the website. CP. = Computed with the provided percentage. AD. = Added during study.

**Table 7:** Data record glossary for PCF-QA dataset (train/test.csv).

Field	Source	Type
Product name	RD.	Text
Question	AD.	Text
Question type	AD.	Text
Question interests	AD.	Text
Evidence index and text	AD.	Numeric and Text
Program	AD.	Numeric and Text
Ground truth answer	AD.	Numeric and Text

PK. = Primary key. FK. = Foreign key. RD. = Raw data in the report. RW. = Raw data from the website. CP. = Computed with the provided percentage. AD. = Added during study.

**Table 8:** Distributions of product metadata fields across companies.

(a) Use location distribution							
Company	ASIA	EU	WW	JP	NA	US	N/A
Dell	—	242 (96.80%)	—	—	—	—	8 (3.20%)
HP	—	—	—	—	440 (100.00%)	—	—
Lenovo	3 (0.33%)	144 (16.00%)	319 (35.44%)	1 (0.11%)	—	429 (47.67%)	4 (0.44%)
Acer	—	—	—	—	—	—	145 (100.00%)
Total	3 (0.17%)	386 (22.25%)	319 (18.39%)	1 (0.06%)	440 (25.36%)	429 (24.73%)	157 (9.05%)

(b) Assembly location distribution					
Company	ASIA	CN	EU	WW	N/A
Dell	3 (1.20%)	238 (95.20%)	1 (0.40%)	—	8 (3.20%)
HP	—	440 (100.00%)	—	—	—
Lenovo	80 (8.89%)	791 (87.89%)	—	14 (1.56%)	15 (1.67%)
Acer	—	—	—	—	145 (100.00%)
Total	83 (4.78%)	1469 (84.67%)	1 (0.06%)	14 (0.81%)	168 (9.68%)

(c) PCF model/standard distribution		
Company	PAIA	ISO 14040 & ISO 14044
Dell	250 (100.00%)	—
HP	—	440 (100.00%)
Lenovo	900 (100.00%)	—
Acer	145 (100.00%)	—
Total	1295 (74.64%)	440 (25.35%)

Abbreviations: ASIA for Asia, EU for Europe, WW for worldwide, JP for Japan, NA for North America, US for the United States, and N/A for not available.

**Table 9:** One record example in the Product CSV file.

Field	Value
Product ID	443
Product name	Latitude 3180
File URL	...
Archive URL	...
Company name	Dell
Use location	US
Assembly location	CN
Product carbon footprinting model/standard	PAIA
Product type	Laptop
Product carbon footprint (kg CO2e)	243
Manufacturing CO2e percentage	85.9
Chassis & assembly CO2e percentage	3.1
HDD CO2e percentage	0
SSD CO2e percentage	21.1
Power supply unit CO2e percentage	7.1
Battery CO2e percentage	2.2
Mainboard and other boards CO2e percentage	26.5
Display CO2e percentage	25.6
Packaging CO2e percentage	0.3
ODD CO2e percentage	—
External components CO2e percentage	—
Others* CO2e percentage	—
Manufacturing CO2e	208.737
Chassis & assembly CO2e	7.533
HDD CO2e	0
SSD CO2e	51.273
Power supply unit CO2e	17.253
Battery CO2e	5.346
Mainboard and other boards CO2e	64.395
Display CO2e	62.208
Packaging CO2e	0.729
ODD CO2e	—
External components CO2e	—
Others* CO2e	—
Validation notes	Automatic verified (within 99%–101% tolerance).

The question-answer records include the product name field for easy access to questions by product name. Additional fields are generated by running our Python scripts on the Product CSV file. The question interests column identifies the component names referred to in the questions and can be used to locate the corresponding ground truth answers. For example, when generating the evidence index, we focus specifically on the components highlighted in the question interests. Since a question may address multiple components, both the ground truth answer and the program’s final answer are represented as lists, maintaining the order in which the components appear. These lists can contain numeric values for percentages or carbon footprints, as well as text values for component names. An example QA record for the same product is shown in Table 10.

**Table 10:** One example record in the QA CSV file.

Field	Value
Product ID	443
Product name	Latitude 3180
Question	What are the carbon footprints of mainboard, batteries, manufacturing, and chassis in the Latitude 3180 laptop?
Question type	Calculation
Question interests	Mainboard, Batteries, Manufacturing, Chassis
Evidence index and text	{“[404,417]”: “243 kgCO2e + / − ”, “[2438,2469]”: “Mainboard and Other Boards 26.5%”, “[2425,2436]”: “Battery 2.2%”, “[2353,2371]”: “Manufacturing 85.9%”, “[2373,2395]”: “Chassis & Assembly 3.1%”}
Program	total_carbon=243.0 mainboard_percent=0.265 mainboard_carbon=total_carbon* mainboard_percent batteries_percent=0.022 batteries_carbon=total_carbon* batteries_percent manufacturing_percent=0.859 manufacturing_carbon=total_carbon* manufacturing_percent chassis_percent=0.031 chassis_carbon=total_carbon* chassis_percent answer=[mainboard_carbon, batteries_carbon, manufacturing_carbon,chassis_carbon]
Ground truth answer	[64.395, 5.346, 208.737, 7.533]

**Fig. 3:**
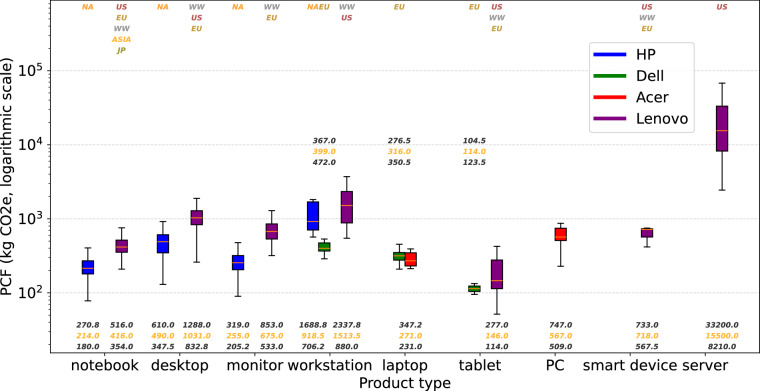
Variations in product carbon footprints across companies and product types.

**Fig. 4:**
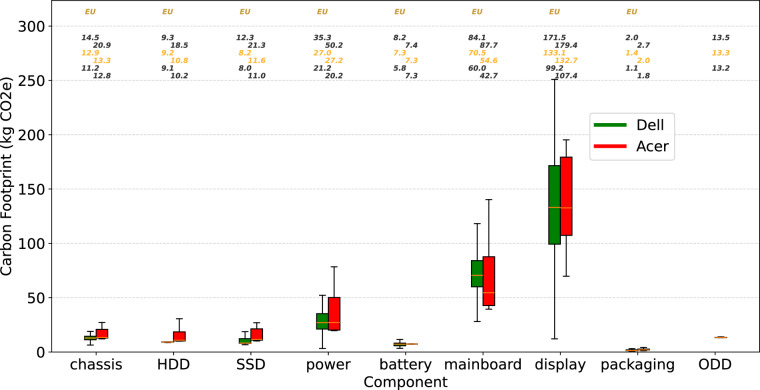
Variations in component-level product carbon footprints.

We analyze the distribution of product metadata fields, as summarized in Table 8. Each cell in the table presents both the product count and the corresponding percentage. Table 8a shows the distribution of product use locations. North America (NA), the United States (US), and Europe (EU) are the three most common use locations. The dominant use location varies across companies: EU for Dell, NA for HP, and US for Lenovo. Acer does not report either the use location or the assembly location in its PCF documents. The distribution of assembly locations is shown in Table 8b, where China (CN) is the most frequent assembly site across all companies. Table 8c presents the distribution of PCF models or standards, with approximately 75% of the reports based on the PAIA model. Because the PAIA model lacks transparency and relies on life cycle assumptions that may be outdated, users should interpret data derived from it with caution. Figure 3 illustrates the variations in Product Carbon Footprints (PCFs) across companies and product types, with the y-axis on a logarithmic scale. Each box summarizes the distribution of product carbon footprints for a company-product type group, with the horizontal orange line inside indicating the median. Gaps between boxes within a product type reflect cases where a company does not offer products in that category. Use locations are displayed at the top of the figure using distinct colors. The ordering is top-down, with the topmost location representing the largest number of products. The three numbers reported for each box correspond to the 25th percentile, median, and 75th percentile. According to Table 8c, Dell, Lenovo, and Acer use the PAIA model, while HP uses ISO 14040 & ISO 14044 standard instead. Therefore, we can only use these three companies for a coarse comparison. Dell and Acer products generally report lower carbon footprints, while Lenovo products tend to have higher values. These differences may result from variations in carbon estimation methods, carbon optimization strategies, and the distribution of product types across companies. PCF also varies across product types. In Fig. 3, we observe that servers, along with desktops and workstations, exhibit a wider range of PCFs and generally higher footprints, as they typically contain a larger number of components. Tablets, on the other hand, have the lowest average PCF, primarily due to their smaller size and fewer components compared to other device categories. For products of the same type, companies show distinct distributions of product carbon footprints (PCFs). Lenovo reports the highest PCFs across most product types. Dell laptops tend to have higher PCFs than Acer’s. Figure 4 illustrates the carbon footprints of individual components of Dell and Acer, with each box summarizing the distribution for a component type of one company and the horizontal line indicating the median. The results show clear variation across components: displays, mainboards, and power supply units generally account for larger shares of total product footprints, while packaging and batteries contribute comparatively little. On average, Acer’s components have relatively higher carbon footprints than Dell’s. Overall, component-level carbon footprints vary widely across computing products. This diversity makes it difficult to establish a general rule that can accurately estimate carbon footprints across all products and companies, underscoring the importance of our dataset in this research field.

## Technical Validation

At each stage of dataset creation (Fig. 2), we implement comprehensive validation measures to ensure data quality and accuracy. The dataset includes a Validation Notes field that documents the validation approach and outcomes for each product. In the data collection phase, we visually inspect the data by generating plots to identify anomalies such as outliers. When outliers are detected, we cross-check the corresponding entries with the original PDF files to manually verify their accuracy. Additionally, we manually review all values that deviate by more than two standard deviations above or below the company median to ensure their correctness. In the question creation phase, we review the grammar of both the question templates and example questions to ensure they are clear and coherent. For the evidence extraction step, we ensure the extracted evidence aligns with the questions being asked. To confirm the accuracy of the ground truth indices, we print the extracted text alongside its corresponding indices from the document and manually verify to check for any errors. In the final step of program generation, all generated programs are executed, and their outputs are compared with the ground truth answers. Programs producing matching results are validated, reinforcing the correctness of the dataset.

We implement programmatic validation to verify the accuracy of the extracted data. This involves summing the manufacturing component percentages and comparing them to either 100% or the claimed manufacturing percentage in the report, depending on the company’s reporting format. For HP, which consistently reports breakdowns summing to 100%, we manually review any samples that deviate beyond a 99%–101% tolerance range. Among 457 samples, 23 fall outside this range. Upon manual inspection, 22 reports were confirmed to have breakdowns that genuinely did not sum to 100%. One exception, the HP E230t 23” Display, was found to have formatting issues in the extracted text that caused an error. The correct percentages were manually retrieved for this report. All validation outcomes are documented in the CSV file’s notes field.

For Dell, the component percentages are compared against the total manufacturing percentage provided in the reports. Of 244 samples, 8 samples were outside the tolerance range. The Latitude 7300 25th Anniversary Edition report used a bar chart for manufacturing percentages alongside a 100% pie chart for the breakdown, leading to discrepancies. We manually corrected the data for this product and resolved pie chart parsing errors in the remaining seven samples. Acer reports underwent the same validation process as Dell, with all records falling within the 99%–101% tolerance range. Conversely, Lenovo reports often use inconsistent pie chart formats, making automated parsing unfeasible. As a result, their component breakdowns were not included in the dataset.

## Usage Notes

In our repository, the datasets directory contains the product and QA datasets, organized into product and QA subdirectories. The product dataset is saved as *products.csv*, while the QA dataset is divided into *train.csv* and *test.csv*. The codes directory includes scripts organized into subdirectories, each named after a step in the dataset creation process as shown in Fig. 2. Unless otherwise noted, all scripts are named with the company name as the primary identifier. First, the data_collection directory contains download and parse subdirectories, which hold scripts for downloading PCF files from company websites and parsing these files, respectively. In download, there are four scripts named *{company name}_download.py*, each designed to download all PDF files from a specific company’s PCF site. Additionally, *download_with_url.py* enables downloading PCF files one by one using URLs listed in *products.csv*. Second, the question_generation directory has four subdirectories corresponding to the four question types in Table 4. Here, *{company name}_pdf.py* extracts raw text from downloaded PCF reports, and *{company name}_pdf_question.py* generates questions using templates. The evidence_extraction directory contains scripts for extracting the evidence needed to answer the generated questions. Lastly, the program_generation directory includes three types of scripts: *{company name}.py* generates programs using templates, *{company name}_gt.py* extracts ground truth answers from *products.csv*, and *{company name}_exec.py* executes the generated programs, comparing results with ground truth to verify program correctness.

## Data Availability

We released our data on figshare^22^. They are under the CC BY 4.0 license (https://creativecommons.org/licenses/by/4.0/). This license allows recipients to freely modify and share the code, promoting widespread application and collaboration.

## Code availability

We released our codes on figshare^22^. They are under the CC BY 4.0 license.
